# Functional, Structural, and Neurotoxicity Biomarkers in Integrative Assessment of Concussions

**DOI:** 10.3389/fneur.2016.00172

**Published:** 2016-10-05

**Authors:** Svetlana A. Dambinova, Joseph C. Maroon, Alicia M. Sufrinko, John David Mullins, Eugenia V. Alexandrova, Alexander A. Potapov

**Affiliations:** ^1^Brain Biomarkers Research Laboratory, DeKalb Medical Center, Decatur, GA, USA; ^2^Neurological Surgery, University of Pittsburgh, Pittsburgh, PA, USA; ^3^Orthopaedic Surgery, University of Pittsburgh, Pittsburgh, PA, USA; ^4^Surgery, Piedmont Hospital, Atlanta, GA, USA; ^5^Neurotrauma, Burdenko Neurosurgery Institute, Moscow, Russia

**Keywords:** concussion, mild TBI, neuropsychological evaluations, advanced MRI sequences, neurotoxicity and neuroplasticity biomarkers

## Abstract

Concussion is a complex, heterogeneous process affecting the brain. Accurate assessment and diagnosis and appropriate management of concussion are essential to ensure that athletes do not prematurely return to play or others to work or active military duty, risking re-injury. To date, clinical diagnosis relies primarily on evaluating subjects for functional impairment using instruments that include neurocognitive testing, subjective symptom report, and neurobehavioral assessments, such as balance and vestibular-ocular reflex testing. Structural biomarkers, defined as advanced neuroimaging techniques and biomarkers assessing neurotoxicity and immunoexcitotoxicity, may complement the use of functional biomarkers. We hypothesize that neurotoxicity AMPA, NMDA, and kainite receptor biomarkers might be utilized as a part of comprehensive approach to concussion evaluations, with the goal of increasing diagnostic accuracy and facilitating treatment planning and prognostic assessment.

## Introduction

Concussion is a heterogeneous injury that requires a multifaceted and comprehensive approach for assessment, diagnosis, and management. Several clinical tools are routinely used, including symptom report, neurocognitive testing, and postural stability/vestibular and oculomotor assessments (1). However, these tools, which represent functional biomarkers, have practical limitations and may not possess adequate sensitivity in diagnosing all concussions. More objective means of assessment, including advanced neuroimaging techniques and neurotoxicity biomarkers, have gained research attention. These may facilitate diagnosis and could potentially assist with monitoring recovery and determining prognosis. Preliminary studies suggest neurotoxicity biomarkers, in conjunction with neurocognitive testing, might improve diagnostic certainty of suspected concussions and may provide valuable information on what domains of brain function are affected (e.g., vestibular system) or the severity of a concussion (2). This may be particularly valuable, since the grading scales for concussion severity are not scientifically validated, lack prognostic utility, and are largely considered antiquated or outdated (3).

Laboratory chemistry tests confirm systemic changes of energy metabolites in concussion (e.g., ATP, creatine, lactate, as well as blood gases and minerals) (4). Quantitative values of brain-borne neurotoxicity degradation fragments of ionotropic glutamate receptors (GluRs) and antibodies to excitotoxicity biomarkers in blood are important early markers of injury (5). Therefore, these biomarkers, when added to a comprehensive evaluation of concussion, may improve diagnosis and prognosis. The purpose of this review is to highlight the potential role of neurotoxicity biomarkers in a comprehensive evaluation of sports-related concussion.

## Clinical Definitions: Concussion

Concussion is defined as a “complex pathophysiological process affecting the brain, induced by biomechanical forces,” with associated traumatically induced alteration in mental status with or without loss of consciousness, as specified in a recent consensus statement on concussion in sports (6). Several physical, cognitive, emotional, somatic, and sleep-related symptoms may be present for days to weeks following injury. These are linked to cognitive, vestibular, and oculomotor dysfunction of various brain systems or domains (6). The terms “concussion” and “mild traumatic brain injury” (mTBI) are proposed to be used interchangeably, since much of the clinical symptomology overlaps and as many as 80% of concussions are diagnosed as mTBI. Neither mTBI nor concussive injuries show gross abnormalities on standard neuroimaging, and most patients recover without permanent impairment (7, 8). Typically, about 80% of post-concussive symptoms resolve spontaneously within 7–10 days of the acute phase (6).

## Biomechanics of Injury

Rotational forces can cause a transient disruption of function in the reticular activating system, resulting in the loss of consciousness associated with concussion (9). Prior biomechanical data have demonstrated that a simple impact of the head with a solid surface (rotational acceleration >5,000 rad/s^2^) leads to the greatest stress–strain to the frontotemporal regions, connecting limbic structures as well as the corpus callosum and cortical–spinal tract (10). As a result, rotational acceleration, diffuse shear, and strain forces cause variable degrees of injury to neurons, glia, the blood–brain barrier (BBB), and vascular structures, leading to transitory ionic functional disturbances with clinical manifestations. These include sudden confusion, lack of balance or coordination, vision abnormalities, and memory impairment (11). Despite using advanced techniques, such as the head impact telemetry system (HITS), researchers have not been able to diagnose concussion reliably by quantifying a specific threshold (12). Researchers argue that the validity of head impact metrics has not been adequately addressed for sports, and clinicians have been cautioned to not rely on impact magnitude or location to predict acute clinical outcomes, symptom severity (Table 1), neuropsychological function, or balance abnormalities (10, 13).

**Table 1:** **Biomechanical attempts to assess severity of concussion**.

Severity	Characteristics		Transitory disturbances		Reference
	Impact force in gravity force (g) and radian per seconds (rad/s^2)^	Impact location	Frequent symptoms	Symptom duration	
Mild	**Direct impact**				
	Linear acceleration ~30–65 g Time ~ 1 ms	Frontal, parietal, and temporal lobes	Often no symptoms, no functional changes	About 24 h	(14)
	Rotational acceleration – 4,000–5,000 rad/s^2^	Brainstem, spinal tract			(15)
	**Coup-countercoup** (AIS^a^ = 1)				(16)
	linear acceleration ~ 50–100 g ICP^b^ < 173 kPa	Frontal lobe and upper end of brainstem			
Moderate	**Direct impact**				
	Linear acceleration ~70–90 g Time ~ 1–3 ms	Frontal, parietal, and temporal lobes	No outward symptoms but substantial functional alterations	1–3 days	(17)
	Rotational acceleration – 5,000–6,500 rad/s^2^	Brainstem, spinal tract			(10)
	**Coup-countercoup** (AIS = 2)				
	linear acceleration ~ 100–150 g ICP ~ 140–190 kPa	Frontal lobe and upper end of brainstem			(18)
Severe	**Direct impact**				
	Linear acceleration >100 g Time – 4 ms	Frontal, parietal, and temporal lobes	Often but not always clinically observed functional impairment	Up to 2–3 weeks	(19)
	Rotational acceleration ~ 7,000–13,000 rad/s^2^	Brainstem, spinal tract			(20)
	**Coup-countercoup** (AIS = 3–4)				
	Linear acceleration ~ 150–250 g ICP ~ 201–282 kPa	Frontal lobe and upper end of brainstem			

*^a^AIS – Abbreviated Injury Scale*.

*^b^ICP – intracranial pressure*.

## Functional Biomarkers: Clinical Assessment

Clinical assessment of concussion is structured around the various domain-specific impairments exhibited following injury – cognitive, vestibular, somatic, and emotional – adapted for various settings, such as on the “sideline” at sporting events as well as during recovery following acute, subacute, and chronic injury (Table 2).

**Table 2:** **Test battery for concussion assessment**.

Tools	Intended use	Properties	Limitations	Reference
**Sideline**
Sports concussion assessment tool (SCAT3)	Sideline assessment diagnostic	Sensitivity 80–94%	Should not be used for return to play decisions	(21)
		Specificity 76–91%		
**Computerized neurocognitive tests**
Immediate post-concussion and cognitive testing (ImPACT)	In office, diagnosis and management	Sensitivity 82–91%	Must have a trained professional for interpretation; potential for misuse (e.g., poor control on environment, use as a standalone measure)	(22, 23) http://www.impacttest.com/
		Specificity 69–89%		
		Test–retest reliability 0.25–0.85		
Automated Neurocognitive Assessment Metrics (ANAM)	In office, diagnosis and management	Test–retest reliability 0.14–0.81		(24) http://www.vistalifesciences.com/index.php/anam-intro.html
Axon Sports Computerized Cognitive Assessment Tool	In office, diagnosis and management	Test–retest reliability 0-0.94 (53% of ICC’s failing minimum standards)		www.axonsports.com/
Concussion Vital Signs	In office, diagnosis and management	Test–retest reliability 0.07–0.87		http://www.concussionvitalsigns.com/
**Postural Stability/Vestibular**
Balance error scoring system (BESS)	Sideline/Acute	Specificity up to 91%	Practice effects, poor intra/inter/after season reliability	(25, 26)
		Sensitivity 34–64%		
Sensory organization test (SOT)	In office	Sensitivity 48–61%		(27)
		Specificity of 85–90%		
Vestibular/oculomotor screening test (VOR, VMS)	In office		Relies heavily on symptom report	(28)
**Oculomotor tests**
Eye-Track Advance (ETA)	Sideline assessment	Sensitivity 54–77%	Depends on past medical history, medications, and drugs used	(29)
		Specificity of 67–92%		
King–Devick (K–D) test	Sideline	Test–retest reliability 0.90	Practice effects, requires baseline	(30)
Vestibular/oculomotor screening test (saccades, near point convergence)	In office	Internal consistency (Cronbach’s alpha = 0.92)	Relies heavily on symptom report	(28)

### Sideline Assessment

The Standardized Concussion Assessment Tool and its revisions (SCAT2, SCAT3) are the most widely used tools on the sideline (31). These comprise a number of empirically validated measures adapted across several domains of assessment, including symptom report, mental status, attention/memory, and balance. The SCAT is “likely to identify the presence of concussion in the early stages of post-injury,” according to the 2013 American Academy of Neurology concussion guidelines (32). Others argue that this tool is insensitive to identifying mild deficits, and is subject to practice effects (33). Furthermore, it should not be relied on for decisions regarding whether an athlete should be allowed to return to play (34). The Display Enhanced Testing for Concussions and mTBI (DETECT) system has been developed in response to the need for a fast and efficient sideline neuropsychological test (35). It is a 7-min battery of tests that allows for real-time cognitive testing in situations previously deemed impractical or unavailable for patients with concussion, such as the sideline. However, it is still being examined for validity and reliability.

### Neurocognitive Testing

Neurocognitive testing has been coined a “cornerstone” of concussion management since the initial Concussion in Sport Group meeting in 2001. Typically, neurocognitive testing is completed in an office or quiet setting, and has been shown to be sensitive both to acute deficits (e.g., <24 h following injury) as well as those detected after an athlete is symptom free (36, 37). Of all the commercially available computerized test batteries, the Immediate Post-concussion and Cognitive Testing Test Battery (ImPACT) is the most widely used and researched and the only one formally approved by the FDA; others are summarized in Table 2. Although largely reserved for those with protracted recoveries, for some athletes, traditional paper and pencil neuropsychological testing may be a component of concussion protocols. While neurocognitive evaluation is essential in assessing and managing concussion in a majority of cases, it is important to recognize that neurocognitive deficits are occasionally absent following concussion. A recent meta-analysis of computerized neurocognitive tests (CNTs) suggests an overall low-to-moderate magnitude of effects size, attributing these findings to the heterogenic nature of concussion (38). There are other limitations in using neurocognitive testing following concussion. Several extraneous factors may influence neurocognitive test performance, including motivation, effort, sleep, pain, and anxiety (39). Neurocognitive testing can be challenging to interpret among subpopulations, such as individuals with learning disabilities or attention deficits in the absence of baseline testing (40).

### Postural Stability/Vestibular

Assessing balance and postural stability may be useful in the acute stages of injury (25). The Balance Error Scoring System (BESS) is the most widely used balance assessment following concussion, due to its rapid ease of administration and low cost. However, the system’s sensitivity has been criticized because of the influence of other factors, such as fatigue, type of sport, and history of ankle injury or instability (41). The Sensory Organization Test (SOT) evaluates dynamics in postural stability using a force plate, and has superior reliability compared to the BESS (27). However, it is not feasible to use on the sideline due to the required equipment. Furthermore, similar to neurocognitive testing, deficits in postural stability are not always present after concussion or may resolve rapidly. Another tool developed for concussion assessment is the vestibular/ocular motor screening (VOMS) (28), which uses several tests for screening central vestibular functioning, including the vestibular–ocular reflex and visual-motion sensitivity.

### Oculomotor Assessment

Both subjective and objective measures of oculomotor functioning have utility in diagnosis and management of concussion. The VOMS measures near point of convergence and provocation of symptoms on saccades, both sensitive to concussion (28), and correlates with neurocognitive impairment (42). The King–Devick (K–D) is a 1-min test in which the athlete is asked to read, quickly and accurately, several pages of single-digit numbers that are arrayed left to right in columns that do not vertically align (43). Preliminary evidence suggests K–D is easily administered, can differentiate concussed from non-concussed athletes, and has high test–retest reliability (*r* = 0.90) (44). However, there is concern regarding practice effects (45). The objective eye-tracking technology concussion test, used in both sports and combat settings, records the subject visually tracking a target as it moves through a circular trajectory (46). Gaze error variability has been found to correlate significantly with attention and working memory measures on neurocognitive testing (29).

## Neuroimaging

Concussion is generally regarded as a functional, rather than a structural, brain injury, with conventional neuroimaging findings considered normal and adding little value to clinical management of this injury beyond ruling out more severe pathology, such as a skull fracture or intracranial hemorrhage (47).

However, advanced magnetic resonance imaging (MRI) methods have demonstrated the ability to detect and localize pathophysiologic consequences of concussion or mTBI (48). Functional MRI (fMRI) data have shown a strong link between brain region functioning vs. concussion severity and time to recovery; however, this procedure is used primarily in the research setting (49). Diffusion tensor imaging (DTI)/DTI-based tractography is used to evaluate axonal tearing after the impact. Quantitative high-resolution imaging techniques, such as susceptibility-weighted imaging (SWI) or angiography (SWAN), depict iron deposits in brain microvessels, while cerebrovascular dysfunction in areas of hyper- or hypoperfusion can be detected by high-resolution diffusion-weighted imaging (DWI).

Recent studies that used SWI or perfusion-weighted imaging (PWI) with DTI and magnetic resonance (MR) spectroscopy found abnormalities in 80% of patients with mTBI when these three imaging modalities were collated (50, 51).

### Diffusor Tensor Imaging

Diffusion tensor imaging (3T DTI)-based tractography evaluates diffuse axonal injury (DAI). Additionally, 3T fluid-attenuated inversion recovery (FLAIR) imaging allows detection of non-hemorrhagic DAI (47) and could potentially assist in assessment of structural abnormalities in concussions. Due to success in assessing a population with moderate-to-severe TBI, DTI has gained popularity for use in mTBI because of its enhanced technical resolution. DTI can reveal white matter alterations affecting the corticospinal tract (CST); the internal capsule; the corpus callosum (52); the inferior/superior, longitudinal, and fronto–occipital fasciculi; and the posterior thalamic and acoustic radiations in athletes following sports concussions (53). While increased fractional anisotropy (FA) numbers indicate reversible changes in white matter, reduced FA values reflect a loss of white matter integrity in a region of interest (54–56). Overall, data from DTI studies of mTBI have been contradictory, however, showing both increased FA values in acute and chronic studies (57, 58) as well as decreased FA levels (59). Recently, Davenport et al. (60) demonstrated that a single football season can produce alterations in DTI parameters in the absence of clinical concussion.

### Diffusion-Weighted Imaging

Microvessel dysfunction can be detected by high-resolution DWI. DWI allows for differentiation between cytotoxic edema (restricted diffusion) and vasogenic edema (increased diffusion), and has been shown to identify shearing injuries not evident on T2/FLAIR or gradient echo sequences (61). Significant involvement of the neurovascular unit has also been shown in pediatric patients with sports-related concussions who experienced persistent symptoms (50). Recent MRI-based studies assessing cerebral flow alterations due to concussions and mTBI are indicating the emerging concept of concussion as a form of cerebrovascular disease (62).

### Susceptibility Weighted Imaging

Small hemorrhages in white matter have been detected by high-resolution SWI in retrospective studies of retired NFL players (63), youths with sports-related concussion (64), and, in amateur boxers, more severe cases of mTBI (65). Significant SWI hypointensity alterations in brains of adult male hockey players within a single season have been observed in the subacute stage of injury compared to baseline images (66). However, SWI does not provide quantitative measures of magnetic susceptibility (67).

### Susceptibility-Weighted Angiography

As with SWI, SWAN focuses on intracerebral small vessels for identifying cerebral hemorrhage and calcifications (68) by visualizing measurable changes in cerebral veins due to hypoxia (69). It is particularly useful in visualizating micro-hemorrhages and lesions near the skull base (70), and has contributed to a proposed MRI grading scale (71, 72). Application of susceptibility-weighted imaging mapping (SWIM) for quantitative assessment (67) of venous blood oxygenation in the acute stage of mTBI has shown an excess of oxygen in impacted areas that may reflect a neuroprotective response following the injury (73).

In summary, several MRI sequences (DTI, DWI, FLAIR, SWI, SWAN) may be valuable in determining the location and level of microstructural abnormalities (micro-lesions) and edema following concussion. However, to date, MRI studies for mTBI are limited by study design, including consensus in methodology; e.g., 1.5 or 3T, sequence, slice thickness, and spatial resolution (74). Furthermore, there is often variability in timing after injury, age of subjects, severity of injury, and brain region analyzed. Additionally, use of advanced imaging-derived techniques, such as DTI, with multi-shell diffusion and high-definition tractography modeling might be useful in identifying subtle structural changes due to mild concussion (52). The development of universal and more inclusive protocol(s) for mTBI studies might help overcome current limitations in pursuit of comparable, reproducible data.

## Glutamater Receptor Biomarkers

Biomarkers for concussion can be categorized as diagnostic or prognostic/monitoring (75, 76). Prospective, diagnostic biomarkers provide information about a disease or condition and may assess the severity of concussion. Prognostic biomarkers (risk assessment) are defined as indicators that may provide the anticipated natural history of a disorder in the absence of a therapeutic intervention. Monitoring biomarkers (recovery) are used to follow a previously diagnosed or established disease or condition; for example, in making a decision whether a person should be returned to play in sports or to work or active military duty.

### Diagnostic Potential

Ideal biomarker(s) for the diagnosis of concussion should (i) reflect the origin of transient subtle brain injury resulting from the impact and show a specific location of subtle brain injury, (ii) include detectable biological markers at an early stage of brain damage (within minutes to hours after event), (iii) correlate with performance on clinical measures, and (iv) demonstrate functional deficits or metabolic disturbances concurrent with advanced neuroimaging (fMRI, DTI, and SWI modalities). Several candidate brain biomarkers are associated with concussion severity (Table 3).

**Table 3:** **Candidate biomarkers for identification of concussions severity**.

Biomarker	Performance characteristics			Strengths (intended use)	Limitations (biomarker studies)	Reference
	Cut off ng/ml	Sensitivity %	Specificity %			
AMPAR peptide	0.4	89–91	91-92	Associated with diffuse axonal injury (DAI)	Need data assessed within 24 h after concussion	(77)
NMDAR peptide	1.0	83	91	A biomarker of microvessel damage and correlates with development of cortical vasogenic edema	Need to be assessed within 24 h after concussion	(5)
Calpain-derived αII-spectrin N-terminal fragment (SNTF)	Not established	100	75	Associated with DAI and shows white matter abnormalities	Low levels in biological liquids for mTBI. There are no concussion studies	(78)
Breakdown products of glial fibrillary acidic protein (GFAP-BDP)	0.03	60–93	75–97	Detects hemorrhage/hematoma and might be used to reduce unnecessary CT/MRI	GFAP-BDP also releases during intracerebral and subarachnoid hemorrhagic strokes. There are no concussion studies	(79)
GFAB autoantibody	2.9–3.0	–	–	Shows dynamic interactions between post-injury and specific autoimmune response	Small sample size of the study	(80)
Kainate receptor peptide	0.5	83–90	83–92	Might be associated with brainstem injury and regulates venous circulation (development of cytotoxic edema)	Need to be assessed within 24 h after concussion	(81)
S-100B	0.06	94	50	A marker of compromised blood–brain barrier	Lack of specificity	(82)
	0.12	29	96			
Total Tau protein	–	Area under curve: 0.91 (95% CI, 0.81-1.00)		Correlates to severity of concussions and predicts longevity of recovery	Small sample size of the study	(83)
UCHL1	–	Area under curve: 0.62 (95% CI, 0.57–0.71)		A potential diagnostic assessment of acute TBI	UCHL1 found in more severe TBI. Limited concussion studies	(84, 85)
		0.72		Associated with outcome		

Neurotoxicity biomarkers include the ionotropic GluRs. The AMPA receptors (AMPAR; GluR1 subtype) are located exclusively in synaptic terminals (77) and could indicate diffuse dendritic–axonal injury (86). AMPAR is primarily distributed in the forebrain and subcortical pathways, including the hippocampus, amygdala, thalamus, hypothalamus, and brain stem (87, 88). These regions of the brain are predictable sources of biomarkers given the functional spatial-temporal coherence, developmental pathways, and cerebral plasticity affected by mild brain injury (89). The NMDA receptors (NMDAR: NR2 subtypes) are localized on the epithelial surface of microvessels that form the BBB and regulate cerebral arterial microvascular function (90). The biomechanical forces that lead to concussion may cause mechanical damage and energy failure in parenchymal cells and endothelia that comprise the BBB. Furthermore, concussion drives neurotoxicity biomarker peptides to be released continuously into the bloodstream through the compromised BBB within hours to days after impact. During the acute phase of concussion, a massive release of glutamate upregulates excitotoxic AMPAR (55, 91). The GluR1-subunit of N-terminal AMPAR fragments are rapidly cleaved by extracellular proteases and released into the bloodstream, where this degradation product, identified as a biomarker of neurotoxicity, can be directly detected (peptide fragment of 5–7 kD).

A feasibility study examining the diagnostic potential of the AMPAR peptide assay was conducted by administering neuropsychological testing (ImPACT) and neuroimaging to concussed athletes (20.8 ± 1.8 years old, 56M/28F, 1–2 weeks post-concussion, GCS = 14–15) and age-, gender-matched healthy controls (21M/19F) in conjunction with measurements of the biomarker (86). The sensitivity (91%) and specificity (92%) of AMPAR peptide to assess acute and semi-acute concussions (preliminary cut off of 0.4 ng/mL) in college athletes was established. Additionally, in athletes with multiple concussions, worse ImPACT scores on processing speed, reaction time, and cognitive efficiency correlated with abnormal levels of AMPAR peptide (2.0–12.0 ng/mL) and DAI changes apparent on MRI (2).

Kainate receptors (KAR, GluR5/6), which are located mostly in the hippocampus, subcortical areas, spinal cord tract, and brainstem (92), might potentially affect cerebral venous circulation. Glutamate serves as a neuromediator for the medulla involved in regulation of involuntary life sustaining functions, such as breathing, swallowing, heart rate, and consciousness (92), primarily through KAR (2). In patients with mTBI, the decrease of venous function due to a rise in venous oxygenation in the affected thalamostriate and right basal areas (93) might involve KAR. As a component of post-military deployment mTBI screening, KAR peptide detection in active duty military personnel (37M/16F, 23.0 ± 1.2 years old, 1 week after blast injury, GCS = 13–15) with impaired venous circulation in cervical areas defined by dopplerography yielded an optimal cut-off value of 1.0 ng/mL (90% sensitivity, 83% specificity), at which a positive predictive value of 93% was achieved. A clinical study conducted with civilians who sustained mTBI (25M/20F, 30.1 ± 3.0 years old, GCS = 13–15) and admitted to ED within 24 h after the impact due to violence-related events, motor vehicle crashes, and incidental falls showed KAR peptide sensitivity of 83% and specificity of 92% (cut-off of 0.5 ng/mL), with a significant positive likelihood ratio of 10.5 to assess severe concussions (unpublished data). Notably, AMPAR and NR2 peptides were also abnormal in these cohorts.

### Prognostic/Monitoring Approaches

Biomarkers intended to measure recovery following concussion should potentially (i) reflect neurological symptoms, (ii) identify the immune system response to inflammation, (iii) further predict outcomes, and (iv) assist with treatment/therapy recommendations.

Disruption of the BBB due to biomechanical force is believed to cause instantaneous increase in brain vasculature permeability, with both immediate and delayed pathogenic consequences (7, 94). The effects of a compromised BBB are exacerbated when accompanied by an immunological response initiated by entry of peripheral anti-CNS autoantibodies (95–97), which may be prognostic biomarkers of brain injury (98). Due to the nature of the normal immune response, antibodies to brain antigens detected in serum are typically associated with advanced or chronic conditions of brain damage (99, 100), whereas neuronal antigens measured in plasma are more likely to reflect acute injuries (101).

Early experimental and clinical research of antibodies to GluR1 subtype of AMPA receptors as an immunoexcitotoxicity biomarker has demonstrated their diagnostic value in detecting pathological brain-spiking activity and epileptic seizures (102, 103) as a consequence of traumatic brain injury, thereby representing a prognostic risk factor. Above threshold increases of AMPAR antibodies measured in serum of children (*n* = 60, 7–16 years old) with chronic posttraumatic headache within 6–12 months after sustained single or multiple concussions were correlated with abnormal brain-spiking activity on EEG (104). Clinical studies of GluR1 antibodies in adult patients with different chronic neurological pathology (*n* = 1866) performed in Russia, Europe, and the USA have demonstrated diagnostic potential (sensitivity of 86–88%, specificity of 83–97%) in assessment of seizures (epilepsy) as consequence of sustained single or multiple concussions (105, 106).

One potential prognostic biomarker is a marked increase in the level of NR2 antibodies. These increased levels are evidence of persistent brain changes related to delayed cerebral ischemia (107), which can follow concussion. In three subjects with prior concussion, NR2 antibody levels remained elevated beyond the cut-off (2.0 ng/mL) throughout the 1.5-year study duration (2). Interestingly, visual memory composite scores on ImPACT were impaired in two of the three athletes. Furthermore, reduction in NR2 peptide and antibodies threshold levels corresponded with improvement on ImPACT scores and reduction of post-concussive symptoms.

An AMPAR peptide assay has been evaluated as a tool for management and decision making for return to play, work, and active military duty following concussion. In a longitudinal study, initial assessment of AMPAR peptide in athletes showed that of 84 subjects (20.8 ± 1.8 years old, 56M/28F, 1–2 weeks post-concussion, GCS = 14–15), 18 (21%) had increased biomarker levels after concussion (108). In the subsequent 15 months, AMPAR peptide values decreased in 11 of the 18 subjects (61%), with levels close to normal (0.07–0.9 ng/mL). These athletes also had normal ImPACT scores, and were allowed to return to play. After an additional 3 months, the seven remaining athletes remained symptomatic and had higher AMPAR peptide values compared to controls. Consequently, they were advised to postpone their return to playing sports and directed to neuroimaging (2).

A recent prospective off-season study assessed AMPAR peptide levels in a professional American football team. Among players (43M, 27.8 ± 3.1) who had no concussions reported in the prior season, AMPAR peptide levels in plasma (within 0.1–1.0 ng/mL) cleared 40 players (93%) to play. The remaining three players (7%) were advised to postpone return to play due to higher AMPAR peptide levels (1.2–2.3 ng/mL). Interestingly, two out of the three players with elevated AMPAR peptide levels reported concussion a week before blood draw, suggesting a cumulative effect. Preliminary assessment of assay performance characteristics in semi-acute (within 2 weeks after the concussion) assessment yielded an 89% sensitivity and 91% specificity (unpublished data).

### Impaired Neuroplasticity Biomarkers for Concussion (Acute and Chronic Conditions)

Due to breakdown of the BBB, most proteins responsible for impaired neuroplasticity enter the bloodstream, fluids, and tissues relatively late (109). Among these are the calpain-derived αII-spectrin N-terminal fragment (SNTF) (78, 110), αII-spectrin breakdown products (SBDPs) for axonal injury (111), S100 associated with compromised BBB (112), astrocyte-specific protein glial fibrillary acidic protein (GFAP), a biomarker of hematoma/hemorrhage (113) and GFAP autoantibody as a post-injury autoimmune response (80), tau protein, a microtubule-associated structural protein located within axons, and ubiquitin carboxy-terminal hydrolase L1 (UCHL1), a cysteine protease expressed in neurons as well as neuroendocrine cells (114) (Table 3).

Following TBI, SNTF accumulates in axons and blood. In a study of patients with mTBI admitted to an emergency room, blood SNTF levels correlated with white matter abnormalities on DTI, as well as cognitive dysfunction and persisted for at least 3 months (110). Among professional ice hockey players, serum SNTF increased at 1 h after concussion and remained significantly elevated from 12 h to 6 days, before declining to preseason baseline. By contrast, serum SNTF levels were unchanged after training in healthy controls (78). Serum SNTF exhibited sufficient diagnostic accuracy for delayed return to play in this study (area under the curve = 0.87).

S100 proteins are small acidic proteins with diverse functions that range from cell cycle progression, transcription protection from oxidative cell damage, and apoptosis (115, 116). A study of collegiate and semi-professional athletes who completed S100B testing at baseline and following concussion demonstrated that relative and absolute increase in serum S100B could accurately distinguish concussion from sport-related exertion (82). A correlation between S100B levels and number of sub-concussive head hits has also been reported (117). Other studies have suggested that low levels of S100B were able to rule out mTBI (118, 119). Recent studies have demonstrated that measurement of S100B would assist in indicating if a CT scan would be needed in the acute setting (120–122). The transient disruption of the BBB from multiple sub-concussive and/or concussive events may allow accumulation of S100B autoantibodies that could be associated with predisposition to neurological disease, including chronic traumatic encephalopathy (CTE). Despite prolonged rest, repetitive head impacts sustained in contact sports have been shown to cause persistent white matter alterations (123). Thus, S100B autoantibodies may be useful biomarker after acute concussion and in evaluating more chronic consequences of head trauma.

Two studies have shown that serum GFAP and its breakdown products (GFAP-BDP), markers of hemorrhage, were increased in patients with an abnormal CT compared to those with a normal CT after mTBI (124, 125). GFAP remained elevated in axonal injury on MRI in a subset of mTBI patients at 3 months’ post-injury (124). Similarly, serum GFAP-BDP was significantly higher in patients with intracranial lesions on CT, compared with those without lesions, and was able to predict which patients required neurosurgical intervention (113). A recent study of 396 patients with mild-to-moderate TBI established that GFAP out-performed S100B in detecting intracranial CT lesions, particularly in the setting of extracranial fractures (126). GFAP-BDP demonstrated very good predictive ability (AUC = 0.87) as well as significant discrimination of injury severity (125). Recent study detecting GFAP autoantibodies found three to five times higher biomarker values in chronic and acute cases accompanies with prior TBI (80). However, it is unclear if serum GFAP would be useful in detecting a more mild form of TBI, such as a sports-related concussion.

Ubiquitin carboxy-terminal hydrolase L1 levels in CSF and serum demonstrated predictive value for severe TBI in adult and children (127, 128) and were also increased after seizures (129). It was suggested that UCHL1 is potentially reporting the BBB integrity (94). Assessments of UCH-L1 after sport-related sub-concussive head hits displayed lack of clinical significance (117).

Tau protein is a microtubule-associated structural protein located within axons. The deposition of hyperphosphorylated tau (p-tau) protein in clusters around small blood vessels of the cortex is indicative of tauopathy and is related to CTE (130). Although the factors that contribute to CTE are poorly understood, both professional athletes (131) and military veterans (132) have been diagnosed with this disease. To date, CTE is primarily diagnosed postmortem (133); however, the first attempt to detect total tau protein in plasma using digital array technology (Table 3) has recently been reported (83).

## Discussion

Clinical assessment tools for concussion all represent functional biomarkers. These include symptom report, neurocognitive testing, and postural stability/vestibular and oculomotor assessments. While widely used to guide clinical care, concern regarding the sensitivity of these functional biomarkers as well as their feasibility for use in the sports arena exists. For example, sideline testing is brief and, therefore, may not be sensitive enough to identify mild concussion. Comprehensive neurocognitive testing may appear normal, leading to premature return to play. More objective means of assessment, including advanced neuroimaging techniques and neurotoxicity biomarkers, have gained research attention. These measures may facilitate diagnosis and potentially assist with monitoring a subject’s recovery. Preliminary studies suggest neurotoxicity biomarkers, in conjunction with neurocognitive testing, may improve diagnostic certainty of suspected concussions as well as provide valuable information on prognosis, allowing a clinician to modify treatment recommendations.

This review highlights potential structural and neurotoxicity biomarkers that could be integrated into the current clinical concussion evaluation that, at present, relies primarily on functional biomarkers. Research to date supports a move toward administering a more comprehensive panel of biomarkers; namely, neurotoxicity markers. These biomarkers represent a second generation of brain “tracers” that could be used to decipher the microstructural abnormalities observed on advanced neuroimaging. For example, abnormal values of one (15A1), two (14AN2, 14NK2, 14 AK2), or three GluRs (12-13ANK3) may assist in making a decision to return a subject to play, extended rest, or further medical assistance (Table 4). Those athletes or patients with abnormal scores using these biomarkers should be directed to neuroimaging assessment (DTI, DWI, SWI, FLAIR), and may benefit from an individualized treatment plan. In the future, it may be possible to classify concussion severity with neuroimaging, similar to proposed MRI classification for TBI (71). Furthermore, the use of neurotoxicity biomarkers may assist radiologists with additional information concerning the area of interest, where microstructural changes might be located (cortical, subcortical, or brainstem injuries).

**Table 4:** **Proposed functional, metabolic, and structural biomarkers to assess severity of acute concussions and mTBI**.

Neuroanatomical area of injury	Clinical and Neuropsychological scores			Neurotoxicity markers^c^	Proposed scores		
	GCS	ImPACT^a^	SCAT3^b^		3T (or higher) MRI^d^		GCS/MRI/biomarkers
					Preferable modality	Scores	
*Subcortical* dendritic-axonal injury (DAI)	15	0.3–0.4	19.3	AMPAR (A)	DTI	1	15A1
*Cortical–subcortical, subcortical–cervical area, or cortical–cervical area, transient events (vasogenic edema)*	14	0.2–0.3	40.7	AMPAR (A) NR2(N) KAR(K)	DWI, DTI	2	14 AN2 –cortical – subcortical
							14AK2 – subcortical –cervical
							14NK2- cortical–cervical
*Cortical-subcortical and cervical area (cytotoxic edema)*	12–13	0.1–0.2	46.2	Overall involvement of ionotropic GluRs ANK	FLAIR, SWI/SWAN, DTI	3	12ANK3
							13ANK3

*^a^Considered only cognitive efficacy score*.

*^b^SCAT3 data were considered from (21)*.

*^c^Considered only abnormal values of AMPAR, AMPA receptor peptide fragment (candidate biomarker of DAI); NR2, peptide fragment of NMDA receptor subtype (arterial microvascular marker candidate); KAR, kainite receptor peptide fragment (venous circulation biomarker candidate)*.

*^d^MRI scores are suggested analog to that proposed by Potapov et al. (71) based on location/level of brain lesions for TBI*.

Accurate diagnosis of concussion and appropriate management of the injury to prevent premature return to play is vital to protecting the long-term brain health of athletes and others at risk of re-injury. Structural and neurotoxicity biomarkers can be used to complement the functional biomarkers currently used to assess concussion to facilitate diagnosis and treatment.

## Human Subjects

This review presents retrospective analyses of neurotoxicity biomarkers measured in subjects with concussions and mild TBI previously disclosed in peer-reviewed scientific publications.

All retrospective analyses of human data concerning GluR biomarker studies conducted by the authors were performed in accordance with institutional and national guidelines and regulations. The institutional review protocol governing the college and professional athletes’ enrollment was approved by the respective Institutional Review Boards at Kennesaw State University, the University of Pittsburgh, and by the USAMRMC ORP HRPO under proposal DM090557 (award W81XWH-11-2-0081). The research protocol for using samples collected from active duty personnel and civilians who suffered mild TBI in the studies described herein were approved by the Ethics Committee at Burdenko Institute of Neurosurgery (Moscow, Russia). Written informed consent was provided by all research subjects. The privacy of participants was protected using global unique identifiers.

## Author Contributions

SD and JDM are joint senior authors. AMS, JDM, EVA, and AAP are contributed equally to this work.

## Conflict of Interest Statement

The authors declare that the research was conducted in the absence of any commercial or financial relationships that could be construed as a potential conflict of interest.
